# Association of microsomal epoxide hydrolase polymorphisms and lung cancer risk

**DOI:** 10.1038/sj.bjc.6601142

**Published:** 2003-08-12

**Authors:** A Gsur, T Zidek, K Schnattinger, E Feik, G Haidinger, P Hollaus, A Mohn-Staudner, C Armbruster, S Madersbacher, G Schatzl, K Trieb, C Vutuc, M Micksche

**Affiliations:** 1 Division of Applied and Experimental Oncology, Institute of Cancer Research, University of Vienna, Austria; 2 Division of Epidemiology, Institute of Cancer Research, University of Vienna, Austria; 3 Department of Surgery, Pulmological Center, Baumgartner Hoehe, Vienna, Austria; 4 Department of Internal Medicine, Pulmological Center, Baumgartner Hoehe, Vienna, Austria; 5 Department of Urology and Andrology, Ludwig Boltzmann Institute for Urological Oncology, Donauspital, Vienna, Austria; 6 Department of Urology, University of Vienna, Austria; 7 Department of Orthopedics, University of Vienna, Austria; 8 Austrian Cancer Society, Vienna, Austria

**Keywords:** lung cancer, mEH, polymorphism, molecular epidemiology

## Abstract

Microsomal epoxide hydrolase (mEH) plays a dual role in the detoxification and activation of tobacco procarcinogens. Two polymorphisms affecting enzyme activity have been described in the exons 3 and 4 of the mEH gene, which result in the substitution of amino acids histidine to tyrosine at residue 113, and arginine to histidine at residue 139, respectively. We performed a hospital-based case–control study consisting of 277 newly diagnosed lung cancer patients and 496 control subjects to investigate a possible association between these two polymorphisms and lung cancer risk. The polymorphisms were determined by polymerase chain reaction/restriction fragment length polymorphism and TaqMan assay using DNA from peripheral white blood cells. Logistic regression was performed to calculate odds ratios (ORs), confidence limits (CL) and to control for possible confounders. The exon 3 polymorphism of the mEH gene was associated with a significantly decreased risk of lung cancer. The adjusted OR, calculated relative to subjects with the Tyr113/Tyr113 wild type, for the His113/His113 genotype was 0.38 (95% CL 0.20–0.75). An analysis according to histological subtypes revealed a statistically significant association for adenocarcinomas; the adjusted OR for the His113/His113 genotype was 0.40 (95% CL 0.17–0.94). In contrast, no relationship between the exon 4 polymorphism and lung cancer risk was found. The adjusted OR, calculated relative to the His139/His139 wild type, was for the Arg139/Arg139 genotype 1.83 (0.76–4.44). Our results support the hypothesis that genetically reduced mEH activity may be protective against lung cancer.

It is estimated that smoking is responsible for about 90% of lung cancer cases. Since not all smokers develop lung cancer, it has been hypothesised that polymorphisms in genes encoding enzymes involved in the metabolisms of tobacco carcinogens such as the microsomal epoxide hydrolase (mEH) may influence an individual's susceptibility to lung cancer.

Microsomal epoxide hydrolase, a phase II metabolic enzyme, catalyses the hydrolysis of arene, alkene and aliphatic epoxides from polycyclic aromatic hydrocarbons and aromatic amines. Although this hydrolysis is generally a detoxification reaction because less reactive and more watersoluble *trans*-dihydrodiols are produced (Oesch, 1973), in the case of some hydrocarbons such as benzo(a)pyrene, present in tobacco smoke, more highly reactive and mutagenic compounds for example the 7,8-diol-9,10 epoxide are generated in the metabolic process (Sims *et al*, 1974). Thus, mEH exhibits a dual role of procarcinogen detoxification and activation (Shou *et al*, 1996) and may be considered a cancer risk factor as well as a protective factor, depending on the carcinogens.

The gene for human mEH, consisting of nine exons, has been mapped with *in situ* hybridisation to chromosome 1q42.1 (1p11-qter) (Hartsfield *et al*, 1998). In the coding region of the mEH gene, two relatively common genetic polymorphisms are characterised within exons 3 and 4. (Hassett *et al*, 1994a, 1994b). In exon 3 of the mEH gene, a C has been substituted for a T, resulting in a tyrosine replacement by histidine at codon 113. *In vitro* expression analyses indicate that this amino-acid change results in a 40–50% decrease in enzyme activity and thus the allele has been called the ‘slow allele’. The second polymorphism occurs in exon 4, a G to A transition, causing a histidine to arginine change at codon 139. This polymorphism has been referred to as the ‘fast allele’ as this change results in a 25% increase of enzyme activity (Hassett *et al*, 1994a).

We conducted a hospital-based case–control study of 277 patients with primary lung cancer and 496 controls without a history of cancer, all of them Austrian Caucasians, to investigate whether the mEH polymorphisms in exons 3 and 4 are associated with lung cancer risk.

## MATERIAL AND METHODS

### Study population

Lung cancer cases were recruited consecutively since January 1997 from the Pulmological Center, Baumgartner Hoehe, Vienna, Austria's largest hospital for the treatment of pulmonary diseases. Cases (*n*=277) were newly diagnosed, previously untreated and histologically confirmed lung cancer patients.

Histological data were available for 255 cases, of those 27.1% had squamous cell carcinoma, 51.0% adenocarcinoma, 4.7% large-cell carcinoma, 14.5% small-cell carcinoma, 0.7% alveolar-cylinder-cell carcinoma and 2.0% mixed histological subtypes. Patients were derived from the Surgical Department (34%) and the Department of Internal Medicine (66%). The hospital-based control group (*n*=496) was recruited at the same time period from the Departments of Urology (41.0%), Orthopedics (15.6%), Gynaecology (11.9%) and Head and Neck (1.9%) of the University Hospital of Vienna; the Department of Internal Medicine (16.8%) and the Department of Surgery (0.8%) of the Pulmological Center; the Department of Urology (1.5%) of the ‘Kaiser Franz Josef Hospital’, an outpatient clinic (8.9%) and relatives of the students involved in this study (1.5%). Only individuals without a history of cancer were eligible for participation as controls. The human subjects protocol for this study was reviewed and approved by the institutional review boards of the participating hospitals. Written informed consent was obtained from all the included subjects.

### Genotyping analyses

Heparinised peripheral blood mononuclear cells (PBMCs) were isolated by Ficoll–Paque gradient centrifugation (Amersham Pharmacia Biotech, Arlington Heights, IL, USA). Genomic DNA was extracted from PBMC, using QIAamp Blood Kit (Quiagen GmbH, Germany).

The mEH polymorphisms in exons 3 and 4 were determined by two separate polymerase chain reaction (PCR)-based methods using primers as described by Harrison *et al* (1999). Briefly, the amplification reactions were carried out in a 50 *μ*l volume consisting of 200 ng genomic DNA, 0.2 *μ*M of each primer (VBC-Genomics, Vienna, Austria), 0.2 mM deoxynuclotide triphosphate (Roche, Vienna, Austria), 1.5 mM MgCl_2_, 5 *μ*l 10 × PCR Buffer II (Applied Biosystems, Norwalk, CT, USA) and 1 U *AmpliTaq* DNA Polymerase (Applied Biosystems). Polymerase chain reactions were performed with a thermocycler 2400 from Applied Biosystems with the following conditions: 94°C for 5 min, followed by 35 cycles of 95°C for 30 s, annealing temperature of 55°C (exon 3) or 62°C (exon 4) for 30 s and 72°C for 30 s, with a final extension of 72°C for 7 min.

A 10 *μ*l aliquot of the appropriate PCR product was digested with 10 U of the restriction enzyme *Eco*RV (exon 3) and *Rsa*I (exon 4) (New England Biolabs, Hitchin, UK) at 37°C for 4 h and separated on a 3% ethidium bromide-stained agarose gel.

The allelic discrimination of the mEH exon 3 polymorphism was reassessed with the ABI PRISM 7000 Sequence Detection Systems (Applied Biosystems), using the fluorogenic 5′nuclease assay with TaqMan Minor Groove Binder (MGB) probes. The wild-type TaqMan MGB probe was FAM labelled (5′-TCAACAGATACCCTCAC-3′) and the mutant probe was VIC labelled (5′-TCAACAGACACCCTC-3′). The final volume for each reaction was 25 *μ*l, consisting of 12.5 *μ*l TaqMan Universal PCR Master Mix (Applied Biosystems), 0.6 *μ*M of each primer (forward primer 5′-CTGGAAGAAGCAGGTGGAGATT-3′ and reverse primer 5′-TCTGGCTGG-CGTTTTGC-3′), 0.2 *μ*M of each TaqMan probe and 100 ng genomic DNA. The PCR profile was an initial denaturation step at 95°C for 10 min and 40 cycles with 95°C for 15 s and 60°C for 1 min, fluorescent signals were measured at 60°C.

Genotyping was done blinded to case–control status. For quality control, 20% of the samples were randomly repeated; no discrepancies were observed.

### Statistical analyses

Univariate statistical analyses included descriptive statistics for age, sex, pack-years, mEH3 and mEH4. Multiple logistic regressions were performed to calculate odds ratios (ORs) and confidence limits (CL) and to adjust for confounders. Confounders included were age, sex and pack-years of cigarettes smoked. Smoking habits were coded as neversmokers and quartiles of pack-years smoked (see Table 2). Deviation of the genotypes from a Hardy–Weinberg equilibrium was assessed using *χ*^2^ statistics. The final multiple logistic regression model was: log(*P*(cancer)/(1−*P* (cancer))=*b*_0_+age+sex+pack-year_quartiles+mEH3+mEH4. To evaluate different histological subtypes, squamous cell carcinomas, adenocarcinomas and small-cell carcinomas were separately analysed using multiple logistic regression and including the same confounders as above. All *P*-values are two-sided; *P*-values <0.05 were considered to be statistically significant. Analysis of the data was performed using the computer software SPSS for Windows (version 10.0).

## RESULTS

In Table 1

**Table 1:** Principal patient characteristics in the study population (*n*=773)

		**Pack-years**	**Age (years)**
**Study population**	**Gender**	**Mean (s.d.)**	**Mean (s.d.)**
Cases (*n*=277)	Female (*n*=88)	44.5 (23.1)	64 (12)
	Male (*n*=189)	55.7 (28.6)	64 (11)
	Total (*n*=277)	52.8 (27.6)	64 (11)
			
Controls (*n*=496)	Female (*n*=160)	22.1 (16.4)	62 (13)
	Male (*n*=336)	36.6 (28.5)	63 (12)
	Total (*n*=496)	33.5 (27.0)	63 (12)
			
All	*n*=773	42.3 (28.9)	63 (12)

selected characteristics of the study population, which consisted of 277 lung cancer patients and 496 controls, are presented. There were no statistically significant differences between the cases and controls in age, mean age was 64 years in the cases and 63 years in the controls. The percentage of males was 68.2% for cases and 67.7% for controls. The means of pack-years for cigarettes smoked showed differences between cases (52.8) and controls (33.5). Furthermore, ORs for lung cancer with respect to quartiles of pack-years are shown in Table 2

**Table 2:** Distribution of quartiles of pack-years of smoked cigarettes in cases and controls and adjusted ORs

**Pack-years (quartiles)**	**Cases (n=277)**	**Controls (n=496)**	**OR (95% CL)**	***P***
0	31 (11.2%)	202 (40.7%)	1	
0.1–22.0	26 (9.4%)	113 (22.8%)	1.97 (1.08–3.57)	0.03
22.0–38.5	51 (18.4%)	83 (16.7%)	5.52 (3.18–9.57)	0.00
38.5–57.7	84 (30.3%)	48 (9.7%)	17.91 (10.01–32.03)	0.00
57.7–288.0	85 (30.7%)	50 (10.1%)	18.07 (10.10–32.34)	0.00

a Adjusted for age, gender, mEH4 and mEH3.

; a highly significant dose–response relationship was found.

The gene frequencies for controls were not in Hardy–Weinberg equilibrium for the exon 3 polymorphism (*P*<0.0001) using the conventional polymerase chain reaction/restriction fragment length polymorphism (PCR/RFLP) genotyping, whereas using a fluorogenic 5′nuclease TaqMan assay they were found to be in Hardy–Weinberg equilibrium (*P*=0.93). The deviation from Hardy–Weinberg equilibrium was attributable to genotyping error, as we found 42 (57.53%) of the heterozygote controls and 19 (54.29%) of the heterozygote patients falsely classified as His113 homozygotes (data not shown). For the statistical analysis, only results performed by the TaqMan assay were used for the exon 3 polymorphism (Table 3

**Table 3:** Distribution of the mEH3 polymorphism in cases and controls and adjusted OR

**Genotype**	**Total (n=773)**	**Cases (n=277)**	**Controls (n=496)**	**OR (95% CL)^a^**	***P***
Wild type	371	147 (53.0%)	224 (45.2%)	1	
Tyr113/Tyr113					
Heterozygous	332	114 (41.2%)	218 (43.9%)	0.77 (0.54–1.09)	0.77
Tyr113/His113					
Homozygous	70	16 (5.8%)	54 (10.9%)	0.38 (0.20–0.75)	0.014
His113/His113					

a Adjusted by age, gender, smoking status (pack-years) and mEH4.

). There was a statistically significant difference in the distribution of genotypes between cases and controls for the exon 3 polymorphism of mEH. The prevalence of the His113/His113 genotype was significantly lower (*P*=0.014) in the lung cancer cases (5.8%) than in the controls (10.9%). The adjusted OR, calculated relative to subjects with the Tyr113/Tyr113 wild type, was 0.38 (95% CL 0.20–0.75). The heterozygous Tyr113/His113 genotype was also lower in the cancer group (41.2%) compared to the control group (43.9%), although the difference was not statistically significant (*P*=0.77).

Table 4

**Table 4:** Distribution of the mEH4 polymorphism in cases and controls and adjusted OR

**Genotype**	**Total (n=773)**	**Cases (n=277)**	**Controls (n=496)**	**OR (95% CL)^a^**	***P***
Wildtype					
His139/His139	552	186 (67.1%)	336 (67.7%)	1	
Heterozygous					
His139/Arg139	224	80 (28.9%)	144 (29.0%)	1.01 (0.69–1.47)	0.95
Homozygous					
Arg139/Arg139	27	11 (4.0%)	16 (3.2%)	1.83 (0.76–4.44)	0.18

a Adjusted for age, gender, smoking status (pack-years) and mEH3.

shows the mEH exon 4 genotype distribution for cases and controls. The gene frequencies for controls were found to be in Hardy–Weinberg equilibrium for the exon 4 polymorphism (*P*=0.905). The adjusted OR, calculated relative to the His139/His139 wild type, was 1.01 (95% CL 0.69–1.47) for the His139/Arg139 genotype and 1.83 (0.76–4.44) for the Arg139/Arg139 genotype, respectively.

An analysis according to histological types and the mEH exon 3 polymorphism was performed, where a statistically significant association for adenocarcinoma was found (Table 5

**Table 5:** Distribution of the mEH3 polymorphism in adenocarcinomas and controls and adjusted ORs

**Genotype**	**Total (*n*=622)**	**Adenocarcinomas (*n*=126)**	**Controls (*n*=496)**	**OR (95% CL)^a^**	***P***
Wildtype	296	72 (57.1%)	224 (45.2%)	1	
Tyr113/Tyr113					
Heterozygous	264	46 (36.5%)	218 (43.9%)	0.63 (0.40–1.00)	0.048
Tyr113/His113					
Homozygous	62	8 (6.4%)	54 (10.9%)	0.40 (0.17–0.94)	0.035
His113/His113					

a Adjusted for age, gender, smoking status (pack-years) and mEH4.

). The adjusted OR, calculated relative to the mEH exon 3 wild type, for the His113/His113 genotype was 0.40 (95% CL 0.17–0.94). Other histological subtypes such as squamous cell carcinoma and small-cell carcinoma revealed no statistically significant association (data not shown).

## DISCUSSION

In this hospital-based case–control study of Austrian Caucasians, we investigated the association between mEH genetic polymorphism and the risk of lung cancer. Our results suggest that the variant genotype of the mEH exon 3 polymorphism is a protective factor for lung cancer, whereas the exon 4 polymorphism of mEH has no influence on lung cancer risk. Using the conventional PCR/RFLP genotyping with primers designed by Harrison *et al* (1999), the alleles for the polymorphism in exon 3 were not in Hardy–Weinberg equilibrium, whereas the alleles of exon 4 polymorphism were found to be in Hardy–Weinberg equilibrium, arguing against a selection bias. This finding is in accordance with other studies using primers designed by Harrison *et al* (1999) for the genotyping of the exon 3 polymorphism. In their Caucasian control population, Zhou *et al* (2001) found genes such as the NADPH quinone oxidoreductase, *N*-acetyltransferase-2 and mEH exon 4 polymorphism in Hardy–Weinberg equilibrium, but not the exon 3 polymorphism. As this finding suggests a misclassification bias, they retested a random of their samples (5%) using different primers and restriction enzymes. However, they found no discordant results, evidently due to the low percentage of their repeats. They conclude that the deviation from Hardy–Weinberg equilibrium of the exon 3 polymorphism requires further studies.

The reverse primer of Harrison *et al* abuts directly at the mutation site and an engineered base change produces an *Eco*RV restriction enzyme site in the wild type. Within this reverse primer, a G → A polymorphism at codon 119 is contained (Yoshikawa *et al*, 2000), which may affect the accuracy of the exon 3 genotyping. Baxter *et al* (2002) found that this primer mismatch in Tyr113/His113 heterozygotes leads to a false His113/His113 genotype when the codon 119 A allele is present. Hence, we reassessed the allelic discrimination of the mEH exon 3 polymorphism with a fluorogenic 5′nuclease assay using TaqMan Minor Groove Binder (MGB) probes. Indeed we found 57.53% of the heterozygote controls and 54.29% of the heterozygote patients falsely classified as homozygotes. In the statistical analysis, only results performed by the TaqMan assay were included.

The variant alleles His113 and Arg139 of the mEH polymorphisms were relatively common, allele frequencies in controls were 0.33 and 0.18, respectively. These frequencies of our control population were comparable to those of previous studies conducted in Caucasian populations, where the frequency of the rare His113 allele ranged from 0.28 to 0.40; of the Arg139 allele from 0.15 to 0.18 (Benhamou *et al*, 1998; London *et al*, 2000; To-Figueras *et al*, 2001).

One of the first studies investigating the association between lung cancer risk and mEH genotypes was conducted by Benhamou *et al* (1998), who found a significant dose–response relationship between predicted enzyme activity and lung cancer risk in French Caucasian smokers. Consecutively, a number of case–control studies have examined the influence of the mEH polymorphisms on lung cancer risk (Persson *et al*, 1999; Lin *et al*, 2000; London *et al*, 2000; To-Figueras *et al*, 2001; Wu *et al*, 2001; Zhao *et al*, 2002; Zhou *et al*, 2001,^2002^), resulting in somewhat inconsistent findings. Recently, Lee *et al* (2002) performed a meta-analysis of seven published studies and a pooled analysis of four published and four unpublished studies to investigate the association of the mEH polymorphisms and lung cancer risk. The results of the meta-analysis indicate no statistically significant association between the two mEH polymorphisms and lung cancer risk. However, in the pooled analysis, comprised of 986 cases and 1633 controls, a significant decrease in lung cancer risk was observed for the exon 3 polymorphic homozygous genotype with an OR of 0.70 (95% confidence interval (CI)=0.51–0.96), adjusted for age, sex, smoking and centre. In contrast, no effect for the exon 4 polymorphism was detectable.

An analysis according to the main histological subtypes, namely adenocarcinoma, squamous cell carcinoma and small-cell carcinoma, revealed a statistically significant association for adenocarcinoma, whereas no significant association was found neither for squamous cell carcinoma nor small-cell carcinoma. This finding is in accordance with the meta-analysis by Lee *et al* (2002), who found the protective effect of the mEH exon 3 polymorphism stronger for adenocarcinoma than for other histological types.

In conclusion, the results of this Austrian Caucasian study support the hypothesis that genetically reduced mEH activity may be protective against lung cancer, especially for adenocarcinoma, as we found a statistically significant reduced risk for individuals homozygous for the exon 3 variant allele. Therefore, the genetic polymorphism at exon 3 of the mEH gene represents a possible mechanism for the modulation of carcinogen disposition that may influence individual susceptibility to cancer development.
